# A Polish and German Population Study of Quality of Life, Well-Being, and Life Satisfaction in Older Adults During the COVID-19 Pandemic

**DOI:** 10.3389/fpsyt.2020.585813

**Published:** 2020-11-17

**Authors:** Ilona Bidzan-Bluma, Monika Bidzan, Paweł Jurek, Leszek Bidzan, Jessica Knietzsch, Marcus Stueck, Mariola Bidzan

**Affiliations:** ^1^Institute of Psychology, University of Gdańsk, Gdańsk, Poland; ^2^Faculty of Health Sciences, Medical University of Gdańsk, Gdańsk, Poland; ^3^Department of Developmental Psychiatry, Psychotic and Geriatric Disorders, Medical University of Gdańsk, Gdańsk, Poland; ^4^DPFA-Academy of Work and Health, Leipzig, Germany; ^5^International Research Academy BIONET, Leipzig, Germany

**Keywords:** anxiety, risk tolerance, quality of life, life satisfaction, well-being, sleep quality, orderly, Pandemic (COVID-19)

## Abstract

**Introduction:** Psychological studies undertaken during the COVID-19 pandemic rarely include people in their 60s or older. In our study, we studied the predictors of quality of life, well-being, and life satisfaction (including risky behavior, trait anxiety, feeling of threat, sleep quality, and optimism) during the pandemic in older people from Germany and Poland and compared them to three different age groups.

**Methods:** A total of 494 adults in four groups−60+ (*N* = 60), 50–60 (*N* = 139), 36–49 (*N* = 155), <35 (*N* = 140)—completed validated self-report questionnaires assessing: socio-demographic data, quality of life, trait anxiety, risk tolerance, Coronavirus threat, optimism regarding the pandemic, difficulty relaxing, life satisfaction, well-being, and sleep quality during the pandemic period.

**Results:** Older people rated their quality of life higher than did young (mean difference=0.74, *SE*=0.19, *p* < 0.01) and middle-aged (mean difference=0.79, *SE*=0.18, *p* < 0.01) participants, rated their life satisfaction higher than young (mean difference=1.23, *SE* = 0.31, *p* < 0.01) and middle-aged (mean difference=0.92, *SE* = 0.30, *p* < 0.05) participants, and rated their well-being higher than young (mean difference=1.40, *SE* = 0.31, *p* < 0.01) and middle-aged (mean difference=0.91, *SE* = 0.31, *p* < 0.05) participants. They also experienced lower levels of trait anxiety and Coronavirus threat (mean difference=-9.19, *SE* = 1.90, *p* < 0.01) than the younger age groups. They experienced greater risk tolerance (mean difference=1.38, *SE*=0.33, *p* < 0.01), sleep quality (*F* =1 .25; *eta*^2^ = 0.01), and optimism (*F* = 1.96; *eta*^2^ = 0.01), and had less difficulty relaxing during the pandemic (*F* = 3.75; *eta*^2^ = 0.02) than middle-aged respondents.

**Conclusions:** Quality of life, life satisfaction, and well-being during the pandemic is affected by age, trait anxiety, and Coronavirus threat. Older people rated their quality of life, life satisfaction, and well-being during pandemic higher than young people, and experienced lower levels of trait anxiety and Coronavirus threat than the younger age groups. They experienced greater risk tolerance, sleep quality, and optimism, and had less difficulty relaxing than middle-aged respondents.

## Introduction

The first case of COVID-19 was reported in Wuhan City, China on the 9th January, 2020 (1), and currently over 8 million cases have been reported in 216 countries (2). The risk of death and serious complications associated with COVID-19 increases with age. Data from most countries indicates that the rise in mortality rates in people suffering from pre-existing medical conditions (severe chronic diseases, e.g., heart disease) was an additional factor burdening the elderly population (3, 4). At the same time, the co-occurrence of other chronic diseases may mask the COVID-19 infection (5).

To define “elderly,” we used the cut-off age of 60 years, as suggested by the WHO (6). Data from the Oxford COVID-19 Evidence Service (from the 25th March 2020) indicates a risk of mortality of 3.6% for people in their 60s, which increases to 8.0 and 14.8% for people in their 70s and 80s, respectively (7). To date, about 80% of COVID-19-related deaths have been of people aged over 60. According to data from the United States, from 10 to 27% people aged over 85 are at risk of death (8, 9).

Apart from the psychological burden associated with the risk of getting infected with a potentially serious and often deadly disease, health authorities in many countries have introduced numerous restrictions that could themselves have had a detrimental effect on the psychological functioning of elderly people (10). The isolation regulations introduced in many countries, including Poland and Germany, limited the availability of many services important for the everyday functioning of elderly people, including medical facilities (which is particularly relevant for elderly individuals with chronic conditions, including mental illnesses). This could have had an adverse effect on their everyday emotional functioning (i.e., cause panic and anxiety) and their cognitive functioning (11).

The restricted contact with other people may have created a sense of danger of loss of social support, which is particularly important for elderly people. Social isolation, especially perceived social isolation (subjective and not necessarily accompanied by a real absence of social life), among older adults heightens their risk of cardiovascular, autoimmune, neurocognitive, and mental health problems (12, 13). Perceived social isolation has a stronger link with mental disorders, especially depressive symptoms (14–17) and neurodegeneration (13, 18).

Fear of COVID-19 has been shown to lead to various anxiety disorders (concerns, panic attacks, insomnia, fear of death, fear of the unknown, PTSD) and depressive states, sustained by the incessant flow of news regarding the virus, the number of infections, mortality rates, and insufficient control, and treatment measures (13). Psychological distress and anxiety [which is a common response to any stressful situation; (19)] impacts sleep quality (20, 21). Reduced sleep quality negatively affects life satisfaction, health status, as well as the social and emotional domains (21–23). Difficulties accessing medical services or specific psychiatric treatment have led to mental relapses and uncontrollable behaviors [hyperactivity, agitation, and self-harm; (24)]. Some researchers report that uncertainty about the possibility of becoming ill and dying and about the health of family and friends has heightened dysphoric mental states (25, 26). On the other hand, social distancing measures slow down the spread of the virus and prevent older people being exposed to the disease (8).

Another consequence of the pandemic was the emergence of widespread reliance on remote technologies; this could be a particular challenge for the elderly. However, because older adults are the least likely to use internet and mobile technologies, they may now experience a greater sense of isolation (11). It has been found that the lockdown and fears about the virus have led to stress in older adults (27). Because older adults are at risk of COVID-19, they are under enormous stress in addition to their existing vulnerabilities. Although the effects of social isolation are different to the effects of loneliness, efforts to reduce social isolation could lead to a lower mortality rate (26, 28).

Until now, the scarce research on the functioning of elderly people during the pandemic has focused on depression, stress, and distress, rather than the positive aspects of quality of life, life satisfaction, and well-being. Furthermore, it has concentrated mainly on those who contracted COVID, e.g., after respiratory rehabilitation (29) or on elderly individuals who are expected to become ill (30). Thus, we decided to include both the positive and negative aspects of the functioning of elderly people during the pandemic, independently of their concerns about falling ill and their health condition at the time of the study.

We understand life satisfaction as an individual's evaluation of their life as a whole, while quality of life refers to the level of general well-being (31–33). Both can be represented on a continuum, but, in the opinion of some researchers [see (34, 35)], life satisfaction is more subjective and can be affected by how a person feels on a given day, whereas quality of life can be measured and fluctuates less. But an individual's own assessment of their quality of life could also be subjective and affected by mood or circumstances (i.e., the current pandemic) and thus similarly variable. Positive well-being has been conceptualized by Ryff et al. (36) and others (37, 38) as subjective (hedonic) well-being, which emphasizes happiness and pleasure, and psychological (eudaimonic) well-being, which focuses on the fulfillment of human potential.

In our study, we focused on identifying the predictors of quality of life, well-being, sleep, and life satisfaction during the pandemic in older people from Central Europe (Germany and Poland), including factors such as risk behavior, trait anxiety, feeling of threat, sleep quality, and optimism, comparing them to three different age groups. This is the first study whose goal was to investigate the psychological functioning of older people during the COVID-19 pandemic and the first study to assess psychological outcomes of the COVID-19 pandemic in older people in Poland and Germany.

## Methods

### Participants and Procedure

The sample comprised 494 adults (72% female, 24% males, 4% missing data) with a mean age of 42.97 years (range 16–82, *SD* = 9.77). Inclusion criteria were: age>18 years and consenting to participate in the study. Exclusion criteria included: age <18 years, not consenting to participate in the study, or no access to the Internet in order to fill-out the study. Table 1 shows the socio-demographic characteristics of the sample. A large majority of the sample were German citizens (80.6%) and the remaining participants were from Poland. Participants were mainly in relationships (58.9%) or single (23.5%).

**Table 1:** Socio-demographic characteristics of the study sample.

**Demographic variable**	**Female (*****n*** **=** **353)**		**Male (*****n*** **=** **120)**		**Total (*****n*** **=** 494)^*^	
	***N***	**%**	***N***	**%**	***n***	**%**
**Age**						
<35	101	28.6	33	27.5	140	28.3
36–49	123	34.8	23	19.2	155	311.4
50–60	95	26.9	35	29.2	139	28.1
60+	34	9.6	29	24.2	60	12.1
**Nationality**						
German	284	80.5	93	77.5	377	80.6
Polish	69	19.5	27	22.5	96	19.4
**Education level**						
Secondary education	147	41.6	52	43.3	208	42.1
University education	206	58.4	68	56.7	286	57.9
**Marital status**						
Single	83	23.5	29	24.2	115	23.3
Married or partnership	208	58.9	69	57.5	295	59.7
Divorced or separated	32	9.1	7	5.8	39	7.9
Widow/widower	1	0.3	–	–	1	0.2
Missing data	29	8.2	15	12.5	44	8.9

* *21 participants did not answer the question about gender, therefore the total sample is not equal to the sum of men and women*.

Most participants were not quarantined either before or during the study (*n* = 378, 76.5%); 83 people quarantined voluntarily and 33 were quarantined in accordance with official procedures. The number of quarantined participants varied across the surveyed age groups (29% for the youngest group, 17% for the middle-aged group, 22% for pre-retirement age, and 28% for older people).

### Research Procedure

The study is a part of broader research project named Health Cube—Survey—Corona Virus COVID19 “Psychological coping, possibilities of crisis intervention and aftercare in companies and institutions for adults, parents and children.” The study was conducted during the pandemic (specifically the period of restrictions between the 27th March and the end of April 2020) in Poland and Germany. The researchers contacted participants by email. The participants completed the surveys online via the link provided. Using Google Forms, a link to a self-report questionnaire was sent by e-mail or made public on other online platforms (e.g., Facebook, Instagram, Messenger, WhatsApp). Participants could contact the researchers via email or other online platforms at any time. The research project was reviewed and approved by the Ethical Committee (decision no. 30/2020) at the Institute of Psychology at the University of Gdansk, Poland. The following research tools were used:

1. A socio-demographic survey created for this study.
2. Quality of life was measured using the mean of an 11-item semantic differential scale (also known as a polarity, polarity profile, or impression differential; 36) consisting of the following items: nervous—free of complaints, confusing—clear, distracted—structured, frightening—fearless, aggressive—peaceful, insecure—self-confident, meaningless—meaningful, helpless—self-controlled, mistrusting—trusting, dependent—autonomous, contradicting—coherent. The short version of the scale was chosen because it measures some features of the long form of the questionnaire more economically. The original version of the semantic differential was developed by Osgood et al. (39) and is used to assess personality attitudes (40). The test respondents are given terms to differentiate between using bipolar scales (39). The given terms should be classified spontaneously rather than rationally and objectively (41). The reliability of the scale in the current study was assessed using Cronbach's alpha, which equaled 0.91.
3. Trait anxiety was measured with the Trait Anxiety Scale (42)—a self-report questionnaire consisting of 10 items (we used the sum of the responses as a measure of the variable). Trait anxiety is the “intraindividually relatively stable, but interindividually varying tendency to perceive situations as threatening and to react to them with an increased state of anxiety, whereby fearful individuals generally react more violently to threatening situations than non-fearful ones” (Krohne, p. 8). The reliability of the scale was assessed using Cronbach's alpha, which equalled 0.84.
4. Risk tolerance was assessed with the single-item Risk Tolerance scale (43): “How do you see yourself – how willing are you in general to take risks?.” Respondents answered on a scale of 1–10 (1-not at all, 10-very much).
5. *The a*uthors' own five single-item measures concerning anxiety related to Coronavirus. Participants were asked to assess the strength of their fears about COVID-19 in relation to: *Coronavirus threat*— “Do you experience the situation regarding the Coronavirus as a threat?” (1-not at all, 10-very much); *Optimism regarding the pandemic—* “Are you optimistic regarding a solution?” (1-very pessimistic, 10-very optimistic); *Difficulty relaxing during the pandemic period*—(“To what extent have you been able to completely relax in calm moments?” (1-without any problems, 10-with great difficulty); *Life satisfaction during the pandemic period-* “How satisfied are you with your life?” (1-not at all, 10-very much); *Wellbeing during the pandemic period—* “How would you describe your state of well-being?” (1-not very good, 10-very good).
6. In addition, the measurement of Coronavirus anxiety levels was supplemented with a single item concerning *Sleep quality during the pandemic period*. Study participants reported their concerns on a five-point Likert scale (1-very bad, 2-bad, 3-medium, 4-good, and 5-very good).
7. Based on known, valid, and reliable measuring instruments, we have modified and developed a single-item scale for measuring general life satisfaction (43, 44).

These single-item scales are economical, valid, and reliable measuring instruments that can reasonably be used for group comparisons in the context of social science surveys if a measurement with more extensive scales is not possible (45). The reliability of these single-item scales was estimated using the test-retest method. In a quota sample with a repeated measurement interval of 6 weeks on average, the stability of the scales was rtt=0.67 (medium stability), which is sufficient for group examinations (45). All measures were in German, so they were translated into Polish and then back-translated. The original items were translated into Polish by two translators independently—a German teacher and a psychologist. Next, the translators settled upon the best Polish version, which was then back-translated (into German) by a Native Speaker who had not seen the original version. A bilingual translator assessed the agreement of the back translation with the original.

### Statistical Analysis

Firstly, we classified respondents into four distinct age groups: young, middle-aged, pre-retirement, and older people. We used theoretical and, statistical criteria to generate these groups in order to give proper meaning to the findings of our study. Respondents in the group of young people were between 16 and 35 years old (*M* = 28.57, *SD* = 4.81); the middle-aged group consisted of people from 36 to 49 years old (*M* = 41.83, *SD* = 3.86); the pre-retirement group ranged from 50 to 60 years old (*M* = 55.17, *SD* = 2.80); and the older group ranged from 61 to 82 years old (*M* = 65.70, *SD* = 5.20).

Then we assessed the means, standard deviations, and intercorrelations (Pearson's r or Spearman's rho depending on the variable's scale) for the study variables on the entire sample. Age differences were assessed by ANOVA by calculating effect sizes. Internal consistency was assessed by calculating Cronbach's alpha coefficients for multi-item scales.

Finally, in order to test the hypothesis regarding the predictors of well-being, sleep, and life quality during the pandemic, we conducted a series of multiple regression analyses using only the sample of older people. Before running the regression analysis, we checked the predictors' multicollinearity using the variance inflation factor (VIF). We used SPSS 26 for all calculations.

## Results

### Descriptive Statistics

Table 2 provides descriptive statistics and correlations of variables examined in the study. It is worth noting that trait anxiety was positively correlated with Coronavirus threat and difficulty relaxing during the pandemic period, while it was negatively correlated with risk tolerance and, all variables regarding quality of life during the pandemic period. The opposite was found for risk tolerance: there was a negative correlation with Coronavirus threat and difficulty relaxing during the pandemic period, and a positive correlation with all variables regarding quality of life.

**Table 2:** Descriptive statistics and intercorrelation matrix for the variables examined in the study.

**Variable**	***M***	***SD***	***1***	***2***	***3***	***4***	***5***	***6***	***7***	***8***	***9***
1. Trait anxiety	34.09	12.65	(0.84)								
2. Risk tolerance (trait)	5.63	2.17	−0.42^**^	–							
3. Coronavirus threat	5.44	2.61	0.29^**^	−0.21^**^	–						
4. Difficulty relaxing	3.98	2.52	0.45^**^	−0.22^**^	0.34^**^	–					
5. Optimism	6.58	2.31	−0.40^**^	0.28^**^	−0.40^**^	−0.35^**^	–				
6. Life satisfaction	7.19	2.02	−0.53^**^	0.28^**^	−0.26^**^	−0.44^**^	0.37^**^	–			
7. Sleep quality	3.56	1.01	−0.47^**^	0.14^**^	−0.14^**^	−0.45^**^	0.26^**^	0.26^**^	–		
8. Wellbeing	7.31	2.05	−0.57^**^	0.25^**^	−0.10^*^	−0.22^**^	0.26^**^	0.37^**^	0.28^**^	–	
9. Quality of life	4.52	1.23	−0.52^**^	0.24^**^	−0.39^**^	−0.52^**^	0.46^**^	0.53^**^	0.30^**^	0.36^**^	(0.91)

*N = 494, alphas on diagonal (for multi-item measures)*.

* *p < 0.05*;

** *p < 0.01*.

### Hypothesis Testing

To investigate the differences between older people and people from other age groups with respect to the variables examined in the study, we conducted an analysis of variance (ANOVA) with the Bonferroni multiple comparisons test. The results show a significant difference among people in different age groups with respect to anxiety as a trait, risk tolerance, difficulty relaxing, life satisfaction, well-being, and quality of life during the pandemic period. The means and standard deviation scores supported with the Bonferroni multiple comparisons test indicated that older people scored less than young people on anxiety (mean difference=-9.19, *SE* = 1.90, *p* < 0.01) and greater than young people on risk tolerance (mean difference = 1.38, *SE* = 0.33, *p* < 0.01). Older people scored less than middle-aged respondents (mean difference=-1.07, *SE* = 0.38, *p* < 0.05) on difficulty relaxing during the pandemic period, and more than young (mean difference=1.23, *SE* = 0.31, *p* < 0.01) and middle-aged (mean difference=0.92, *SE* = 0.30, *p* < 0.05) respondents on life satisfaction during the pandemic period; they scored more than young (mean difference=1.40, *SE* = 0.31, *p* < 0.01) and middle-aged (mean difference=0.91, *SE* = 0.31, *p* < 0.05) respondents on well-being during the pandemic period, as well as more than young (mean difference=0.74, *SE* = 0.19, *p* < 0.01) and middle-aged (mean difference=0.79, *SE* = 0.18, *p* < 0.01) participants on quality of life during the pandemic period. Descriptive statistics for the sample and ANOVA results are presented in Table 3.

**Table 3:** Age differences among variables examined in the study.

**Variable**	**Young**	**Middle-aged**	**Pre-retirement**	**Older**	***F***	***eta^**2**^***
	**M (SD)**	**M (SD)**	**M (SD)**	**M (SD)**		
Trait anxiety	38.34 (12.62)	35.00 (13.12)	30.88 (11.81)	29.15 (9.61)	12.20^**^	0.07
Risk tolerance (trait)	4.94 (2.10)	5.63 (2.10)	6.01 (2.17)	6.32 (2.12)	8.45^**^	0.05
Coronavirus threat	5.11 (2.47)	5.63 (2.56)	5.62 (2.82)	5.32 (2.59)	1.29	0.01
Difficulty relaxing	3.95 (2.57)	4.49 (2.72)	3.70 (2.32)	3.42 (2.14)	3.73^*^	0.02
Optimism	6.49 (2.40)	6.29 (2.37)	6.91 (2.15)	6.78 (2.23)	1.96	0.01
Life satisfaction	6.74 (2.25)	6.94 (1.87)	7.63 (1.93)	7.86 (1.72)	7.68^**^	0.05
Sleep quality	3.45 (1.06)	3.53 (1.09)	3.65 (0.90)	3.67 (0.93)	1.25	0.01
Wellbeing	6.69 (2.20)	7.17 (2.05)	7.75 (1.94)	8.08 (1.44)	9.86^**^	0.06
Quality of life	4.37 (1.08)	4.32 (1.21)	4.65 (1.38)	5.11 (1.06)	7.31^**^	0.04

*N = 494, df = 3*.

* *p < 0.05*;

** *p < 0.01*.

*The differences remain significant even after excluding quarantined individuals (see Supplementary Table 1)*.

In order to test predictions regarding the effects of risk tolerance, trait anxiety, difficulty relaxing, and optimism (controlling for age, sex, education level, and being quarantined) on dependent variables, four separate multiple regression analyses were run. The variance inflation factors estimated for predictors included in the models did not show the multicollinearity problems (VIF ranged from 1.04 to 1.84). For estimating regression coefficients and standard errors, we applied the bootstrap procedure with 1,000 samples. A summary of the results of these analyses is presented in Table 4.

**Table 4:** Summary of results of multiple regression analyses.

**Predictor**	**Life satisfaction during the pandemic period**		**Well-being during the pandemic period**		**Quality of life during the pandemic period**		**Sleep quality during the pandemic period**	
	**B [95% C.I.]**	**SE**	**B [95% C.I.]**	**SE**	**B [95% C.I.]**	**SE**	**B [95% C.I.]**	**SE**
Age	−0.04 [−0.12;0.06]	0.05	0.04 [−0.06;0.10]	0.04	−0.04 [−0.09;0.02]	0.03	−.01 [−0.05;0.04]	0.02
Sex	0.13 [−0.63;0.96]	0.41	0.28 [−0.49;0.90]	0.35	−0.10 [−0.61;0.41]	0.25	0.03 [−0.49;0.73]	0.26
Education level	−0.38 [−1.15;0.43]	0.40	−0.92^**^ [−1.45; −0.33]	0.28	−0.31 [−0.77;0.16]	0.24	0.07 [−0.45;0.73]	0.30
Quarantine	0.81 [−0.05; 1.57]	0.42	−0.31 [−0.96;0.36]	0.36	−0.36 [−1.01;0.27]	0.34	0.07 [−0.58;0.66]	0.31
Risk tolerance	0.03 [−0.12;0.25]	0.10	0.08 [−0.08;0.23]	0.08	0.04 [−0.10;0.15]	0.07	0.13 [−0.02;0.28]	0.08
Trait Anxiety	−0.08^**^ [−0.12; −0.03]	0.02	−0.06^**^ [−0.09; −0.03]	0.02	−0.04^**^ [−0.06; −0.02]	0.01	−0.02 [−0.05;0.01]	0.01
Coronavirus threat	0.07 [−0.13;0.24]	0.09	0.13 [−0.01;0.26]	0.07	−0.04 [−0.15;0.07]	0.05	0.00 [−0.15;0.14]	0.07
Difficulty relaxing during the pandemic period	−0.25^*^ [−0.46; −0.03]	0.11	−0.23^*^ [−0.45; −0.07]	0.10	−0.08 [−0.15;0.06]	0.06	0.05 [−0.11;0.22]	0.08
Optimism regarding the pandemic	0.10 [−0.12;0.30]	0.11	0.12 [−0.03;0.28]	0.08	0.11 [.01;0.26]	0.06	−0.05 [−0.22;0.12]	0.09
**Adjusted** ***R***^**2**^	**0.39**		**0.44**		**0.47**		**−0.04**	

*The bold value indicates, B - unstandardized regression coefficient; C.I. - confidence interval; SE - standard error. N = 60*,

* *p < 0.05*,

** *p < 0.01, bootstrap results are based on 1,000 bootstrap samples*.

The results of the four multiple regression analyses showed a significant and negative effect of trait anxiety on three of the tested variables: life satisfaction (*B* = −0.08, *SE* = 0.02, β = −0.47, *p* < 0.01), well-being (*B* = −0.06, *SE* = 0.02, β = −0.39, *p* < 0.01), and quality of life (*B* = −0.04, *SE* = 0.01, β = −0.36, *p* < 0.01) during the pandemic period (specifically, during the restriction period from the 27th March until the end of April), indicating that life satisfaction, well-being, and quality of life during the pandemic period were lower for older people with high anxiety. Additionally, we found that difficulty relaxing during the pandemic period was a significant, negatively correlated predictor of life satisfaction (*B* = −0.25, *SE* = 0.10, β = −0.31, *p* < 0.05) and wellbeing (*B* = −0.23, *SE* = 0.10, β = −0.34, *p* < 0.05).

## Discussion

Quality of life research in the 60+ age group during the pandemic suggests contradictory results. In our research, older people rated their quality of life, well-being, life satisfaction, and quality of sleep better than all three of the younger comparison groups. Vietnamese studies (30) show that during the COVID-19 pandemic, older people had lower quality of life than the younger age groups. Numerous studies, such as Huong et al. (46), have indicated associations between lower quality of life and older age (≥80 years), and lower education levels. In our opinion, the higher assessment of quality of life, well-being, and life satisfaction in the elderly people who took part in our study might be associated with both their education (most of them reported University education, i.e., 61.7%—which is more than in the total sample) and the financial stability (most of them with the right to retirement) of the elderly people in Poland and Germany during the pandemic. In contrast to younger individuals, people receiving retirement pensions were not facing the threat of job loss. This is supported by the existing literature, which indicates that higher levels of quality of life among people aged 60 years and older depend on factors broadly ranging from socioeconomic status to overall health and the ability to maintain an active and independent lifestyle (47, 48). Here, it is worth stressing that research indicates that older people perceive “successful aging” as positive when associated with the absence of illness and the experience of positive reinforcements in the areas of activity, income, social life, and the relationship with one's family (49). Creative and social activities that sustain belonging to a social group support the positive aging process (50). The higher assessment of quality of life, well-being, and life satisfaction in the studied sample of elderly people is also associated with lower anxiety.

The result indicating a lower level of perceived anxiety and Coronavirus threat in older people is interesting because this group is exposed to the greatest risk of developing COVID-19. Asian studies show that, during the COVID-19 pandemic, 37.1% of elderly people experienced depression and anxiety. Moreover, Qiu et al. (51) have recently indicated that the emotional reaction of older people aged (over 60 years old) is more pronounced. The study found gender differences in this emotional response, with women experiencing more anxiety and depression than men. However, in our study, gender was not a significant predictor of quality of life, well-being, or life satisfaction.

The results of our research indicate lower anxiety levels and Coronavirus threat levels in older people, which can depend on many variables. One explanation may be their limited access to news (beyond radio and TV) that could increase their awareness of COVID-19 and thus affect their level of anxiety. In addition, some studies indicate that despite the existence of COVID-19 measures and, in spite of the lockdown, some older people self-isolated less than others (52) because they needed to look after their grandchildren (while the nurseries, kindergartens, and schools were closed), which may have had a positive effect on their mood.

Relatively speaking, during a pandemic, older people may have the least to lose compared to younger people, who are afraid of losing their social status and jobs as well as not being able to provide for their families, as they generally have a well-established professional position and/or receive a pension. Throughout their lives, older people have experienced various crises. Some individuals from both countries are World War II survivors, but all older respondents grew up in the shadow of WWII, because their parents or grandparents experienced it; the same applies to Martial Law (13th December 1981–22nd July 1983) in Poland, and the erection (13th August 1961) and the fall of the Berlin Wall (9th November, 1998) in Germany. These experiences could have taught them to remain more detached from the news, but may have also given them the sense that—in the words of one of the participants— “one can live through anything.” These experiences might have affected their perception of the COVID-19 pandemic. In addition, older people often compare themselves mainly with people from their own age group, who are often in a worse position (47).

A factor that seems to have a positive impact on life satisfaction is sleep. Recognized as an important element of human life, it strongly affects our emotional states. In addition, short sleep duration and poor sleep quality have a significant impact on lower life satisfaction levels (22, 53). Results indicating better quality of sleep (which is closely related to lower anxiety) in older participants may also explain higher life satisfaction. The results indicate a significant dissimilarity between people in different age groups with respect to trait anxiety, risk tolerance, difficulty relaxing, life satisfaction, well-being, and quality of life during the pandemic period.

In our research, older people had greater risk tolerance than young people. This result contradicts the findings of research indicating that risk appetite and the tendency toward risky behavior decrease with age (54, 55). However, it is worth noting that people differ systematically in their risky behavior, and risk avoidance, and willingness to take risks (56). This personality trait plays an important role in the COVID-19 crisis, because it influences steps taken to protect one's own health and the extent to which people put themselves in danger, for example, by disregarding rules (e.g., not wearing a face mask, ignoring social distancing rules). Empirical findings support the assumption that self-reported willingness to take risks is a personality trait that changes over time and depends on the situation and context (56).

Relationships between willingness to take risks and satisfaction with life (54, 57) and self-efficacy (58, 59) are also reported. People who describe themselves as highly willing to take risks often tend to behave in a risky manner (56). The life experience and personal development of older people may indicate that, for them, taking risks during the pandemic, and thus increasing self-efficacy, is necessary. Older people had less difficulty relaxing during the pandemic than middle-aged people. This is probably related to the previously described economic and professional stability and the greater occurrence of risky behavior.

In comparison to the three youngest groups of participants, older people felt greater optimism regarding the pandemic. Only the group of participants aged 50–60 was more optimistic than the oldest group, which may be associated with a more realistic approach to life than that of young people. Ferguson and Goodwin (60) found that optimism is a predictor of both subjective and mental well-being, while the perception of control (in our research, risk behavior) mediates the relationship between optimism and psychological well-being. Dispositional optimism has been defined as the generalized expectation that a person will obtain good outcomes in life (61). It is construed as a stable personality characteristic. The positive effects of optimism have been demonstrated across diverse, stressful situations (53, 62). The positive effects of optimism could be mediated through positive coping strategies, for example, optimists use more problem-focused strategies—information seeking and positive reframing (62). Many researchers indicate that younger adults are more optimistic than older adults about their own future in 15 years. In contrast, in Durbin et al. (63), both age groups were similarly optimistic about their future at age 85 and expected it to be more positive than others' futures at this age.

This result indicates that the elderly people were more optimistic during the COVID pandemic, which could be explained by the lower number of potential stressors—for instance those associated with potential job loss, which was common among young individuals (64). This could have translated into lower anxiety, which is associated with higher optimism (62).

### Strengths

The main strength of this study was that the research was carried out in a strictly defined time frame, during the pandemic period (specifically the restrictions between the 27th March and the end of April 2020) in Central Europe in two countries (Poland and Germany), on a fairly large population with varied age, sex, and socioeconomic status. The study concerned risk factors negatively affecting quality of life, life satisfaction, and well-being, as well as protective factors, improving the assessment of the respondents' psychological state during the pandemic.

Another strength of this study is the use of simple, short, and easy-to-comprehend scales measuring various constructs, which means high efficiency at low cost. Overall, the single-item scales are economical, valid, and reliable measuring instruments that can reasonably be used for group comparisons in the context of social science surveys, if measurement with more extensive scales is not possible (45). The inclusion of various age groups and showing the determinants of quality of life, well-being, sleep, and life satisfaction of elderly people compared with other age groups are also strengths of this study.

### Limitations

The limitations of this study include the fact that quality of life, life satisfaction, and well-being were restricted to a few selected factors. The sample of elderly people is not representative because it is more likely for older individuals to not be familiar with new technologies. The presented research deals with people who are able to use such technologies, and therefore also have more ways to stay in touch with others, are more informed etc. This could have influenced the results. People who do not use digital media (computer/mobile with online access) could not participate in the study. The elderly people who took part in this study were more likely to report higher education. Because older individuals are usually less likely to use digital media, we expected that this sample will be smaller than the sample of young individuals, and thus we could not ensure that the sample is representative in terms of education and profession. Another limitation of this study is the low number of participants aged more than 60 years (<20% of the overall sample, but we are comparing the elderly population with different age groups, so this could be a strong point of the research, too), as well as the low percentage of Polish participants. We were unable to include in this study all the variables which could affect quality of life, well-being, sleep, and life satisfaction in older adults during the COVID-19 pandemic, e.g., socioeconomic status.

## Conclusion

The findings show that quality of life, life satisfaction, and well-being during the pandemic are affected by the respondent's age, trait anxiety, and Coronavirus threat. Older people rated their quality of life, life satisfaction, and well-being during the pandemic higher than young people and experienced lower levels of trait anxiety and Coronavirus threat compared to younger age groups. They experienced greater risk tolerance, sleep quality, and optimism regarding the pandemic and had less difficulty relaxing during the pandemic than middle-aged respondents.

In summary, it is worth noting that despite the better psychological functioning of older adults in comparison to young adults during the pandemic, it is necessary to implement various forms of help to improve the psychological resources encouraging quality of life in older people, including stress reduction methods which focus on the body, such as breathing meditation and Autogenic Training (65–68), as well as methods based on cognitive behavioral therapy (69).

## Data Availability Statement

The raw data supporting the conclusions of this article will be made available by the authors, without undue reservation.

## Ethics Statement

The studies involving human participants were reviewed and approved by Ethical Committee (decision no. 30/2020) at the Institute of Psychology at the University of Gdansk, Poland. The patients/participants provided their written informed consent to participate in this study.

## Author Contributions

IB-B: conceptualization, project administration, methodology, formal analysis, writing, and original draft preparation. MB: conceptualization, methodology, formal analysis, writing, original draft preparation, and supervision. PJ and LB: conceptualization, methodology, formal analysis, and writing. JK: conceptualization, investigation, and project administration. MS: conceptualization, methodology, formal analysis, writing, and supervision. All authors contributed to the article and approved the submitted version.

## Conflict of Interest

The authors declare that the research was conducted in the absence of any commercial or financial relationships that could be construed as a potential conflict of interest.
