# Supplementary material for: A Polish and German Population Study of Quality of Life, Well-Being, and Life Satisfaction in Older Adults During the COVID-19 Pandemic
Source: Front Psychiatry. 2020 Nov 17;11:585813. doi: 10.3389/fpsyt.2020.585813 (PMC7705096; doi:10.3389/fpsyt.2020.585813)
Supplement: Supplementary file 1 [file Table_1.docx]

**Supplementary material**

Table S1. Age differences between variables examined in the study (excluding people in quarantine)

| Variable | Young | Middle-aged | Pre-retirement | Older | F | eta^2^ |
| --- | --- | --- | --- | --- | --- | --- |
|  | M (SD) | M (SD) | M (SD) | M (SD) |  |  |
| Trait anxiety | 36.81 (11.67) | 34.56 (13.22) | 29.68 (11.58) | 28.84 (10.32) | 8.38** | .06 |
| Risk tolerance (trait) | 5.23 (2.10) | 5.59 (2.09) | 6.05 (2.20) | 6.42 (2.05) | 4.30** | .03 |
| Coronavirus threat | 5.04 (2.46) | 5.59 (2.56) | 5.78 (2.73) | 5.60 (2.61) | 1.52 | .01 |
| Difficulty relaxing | 3.68 (2.36) | 4.45 (2.76) | 3.76 (2.42) | 3.42 (2.19) | 2.91* | .02 |
| Optimism | 6.71 (2.28) | 6.41 (2.44) | 7.05 (2.14) | 6.98 (2.30) | 1.69 | .01 |
| Life satisfaction | 6.96 (2.09) | 6.99 (1.82) | 7.79 (1.81) | 7.67 (1.89) | 5.11** | .04 |
| Sleep quality | 3.62 (.99) | 3.47 (1.10) | 3.70 (.92) | 3.65 (.95) | 1.11 | .01 |
| Wellbeing | 6.96 (2.23) | 7.16 (2.02) | 7.88 (1.87) | 8.19 (1.42) | 6.54** | .05 |
| Quality of life | 4.49 (1.02) | 4.33 (1.22) | 4.65 (1.40) | 5.24 (1.03) | 6.48** | .05 |

*N*=376, *df*=3, **p*<.05; ***p*<.01.
